# Corrosion and Tribology of Materials Used in a Novel Reverse Hip Replacement

**DOI:** 10.3390/ma10070751

**Published:** 2017-07-05

**Authors:** Linda Braddon, Zafer Termanini, Steven MacDonald, Jay Parvizi, Jay Lieberman, Victor Frankel, Joseph Zuckerman

**Affiliations:** 1 Secure BioMed Evaluations, Woodstock, GA 30188, USA; lgb@securebme.com; 2 Hip Innovation Technology, Boca Raton, FL 33433, USA; GeneralInquiries@HIT-IRH.com; 3 London Health Sciences Centre, University Campus in London, London, ON N6A 5A5, Canada; steven.macdonald@LHSC.ON.CA; 4 Sidney Kimmel Medical College, Rothman Institute at Thomas Jefferson University Hospital Philadelphia, Philadelphia, PA 19107, USA; javadparvizi@gmail.com; 5 Department of Orthopaedic Surgery, Keck USC School of Medicine, Los Angeles, CA 90033, USA; jay.lieberman@med.usc.edu; 6 Department of Orthopaedic Surgery, New York University Hospital for Joint Diseases; New York, NY 10003, USA; victorfrankel6@gmail.com

**Keywords:** hip arthroplasty, reverse design, corrosion, wear debris

## Abstract

Total hip arthroplasty has been utilized for the past 50 years as an effective treatment for degenerative, inflammatory and traumatic disorders of the hip. The design of these implants has generally followed the anatomy of the hip as a ball and socket joint with the femoral head representing the ball and the acetabulum representing the socket. We describe a novel hip arthroplasty design in which the “ball” is located on the acetabular side and the “socket” is located on the femoral side. The results of extensive biomechanical testing are described and document wear and corrosion characteristics that are at least equivalent to standard designs. These results support clinical assessment as the next step of the evaluation.

## 1. Introduction

The approach to total hip arthroplasty has generally utilized the same materials and geometric combinations since the original design was introduced by Sir John Charnley [1]. There have been some innovations with respect to processing of polyethylene but, generally speaking, the general design and combinations of materials have remained unchanged. The purpose of this paper it to evaluate a novel reverse design for a total hip arthroplasty. This reverse hip system consists of an acetabular component (acetabular cup and articulating ball), a femoral stem, and a polyethylene lined femoral cup. In this design, the “ball” is on the acetabular side and the “socket” is on the femoral side. The geometry of the reverse hip prosthesis includes two tapers: the first connecting the acetabular ball and acetabular shell and the second taper connecting the femoral cup and femoral stem. The motivation for the development of a reverse hip arthroplasty system was to offer innovative solutions to typical short-comings of conventional hip systems. For example, the aging population indicated for hip replacement surgery is typically more physically active that previous patient populations leading to an increased demand for device stability even in demanding physical activities. The reverse hip evaluated in this study offers enhanced range of motion without jeopardizing stability. The work presented in this paper presents some of the non-clinical testing performed on the device to support the claim of safety and efficacy for its intended use. 

As with all hip replacement systems, there are several non-clinical tests which are critical in evaluating the mechanical stability, the potential for fretting corrosion and wear debris generation. For this analysis, ISO 7206-6 is one of the gold standards for in vitro hip testing and was used to evaluate dynamic fatigue of the system and fretting corrosion of the tapers of the reverse hip system after 50 million cycles (million cycles) [2]. ISO 7206-6 testing is typically performed to evaluate fatigue performance characteristics, however when performed in saline at physiologically relevant temperatures, this testing can also be a reliable indicator of fretting potential. Additionally, ISO 14242 [3] was used to perform a comparative evaluation of the wear characteristics of the hip systems with malpositioned acetabular components for 5 million cycles (million cycles) and an additional 1 million cycles with 2 mm of gate separation. A 2 mm separation was chosen based on a review of published literature. Specifically, Lombardi et al. reported an average separation of 1.2 mm in evaluated THA systems [4]. Additionally, Blumenfeld et al. reported an average separation of 1.53 mm during pivoting activities [5]. Based on these studies, it seemed reasonable to further challenge the novel reverse device with a 2 mm separation. The endpoints of this evaluation included both generation of wear debris and potential for subluxation resulting in metal on metal contact with the articulating portions of the device.

The goal of this evaluation was to determine the wear and corrosion characteristics of this novel hip implant system which uses similar materials such as titanium, cobalt-chrome and highly crosslinked polyethylene as conventional systems but in a different configuration. The testing described is an essential next step in progressing to the clinical use of this novel hip arthroplasty system.

## 2. Materials and Methods 

### 2.1. Device Description

**Figure 1:**
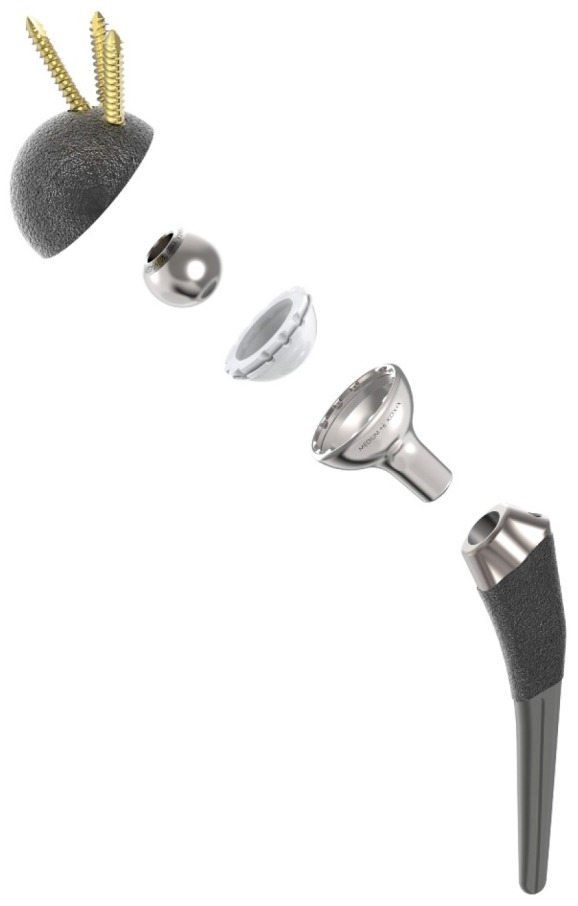
Hip Innovation Technology (HIT) Hip Replacement System (HRS).

The Hip Innovation Technology (HIT) Hip Replacement System (HRS) is designed to enhance stability and reduce the risk of dislocation when compared to current, conventional Total Hip Arthroplasty (THA) systems. In addition, the system has been designed to improve overall contact between articulating surfaces to reduce edge loading and subsequently reduce wear. Like all conventional hip replacement systems, the HRS consists of a femoral stem, an acetabular cup, a cobalt-chrome ball, and a highly crosslinked high molecular weight polyethylene liner. What makes the HRS unique from currently available hip implants is the implementation of a reverse geometry, as shown in Figure 1. With the HIT HRS, a cobalt-chrome ball is fixed to the acetabular cup and a polyethylene lined femoral cup is attached to the femoral stem; standard THA systems have the polyethylene liner attached to the acetabular cup and the cobalt-chrome ball attached to the femoral stem. The polyethylene lined femoral cup now glides around the fixed acetabular ball. By reversing the geometry, the HIT HRS maintains greater contact area between the acetabular and femoral components. This increased contact area enables the HRS to provide enhanced stability, even at extended ranges of motion, in all directions with minimal risk of dislocation. The system is designed for use without bone cement in total hip arthroplasty. 

### 2.2. Corrosion at the Tapers

**Figure 2:**
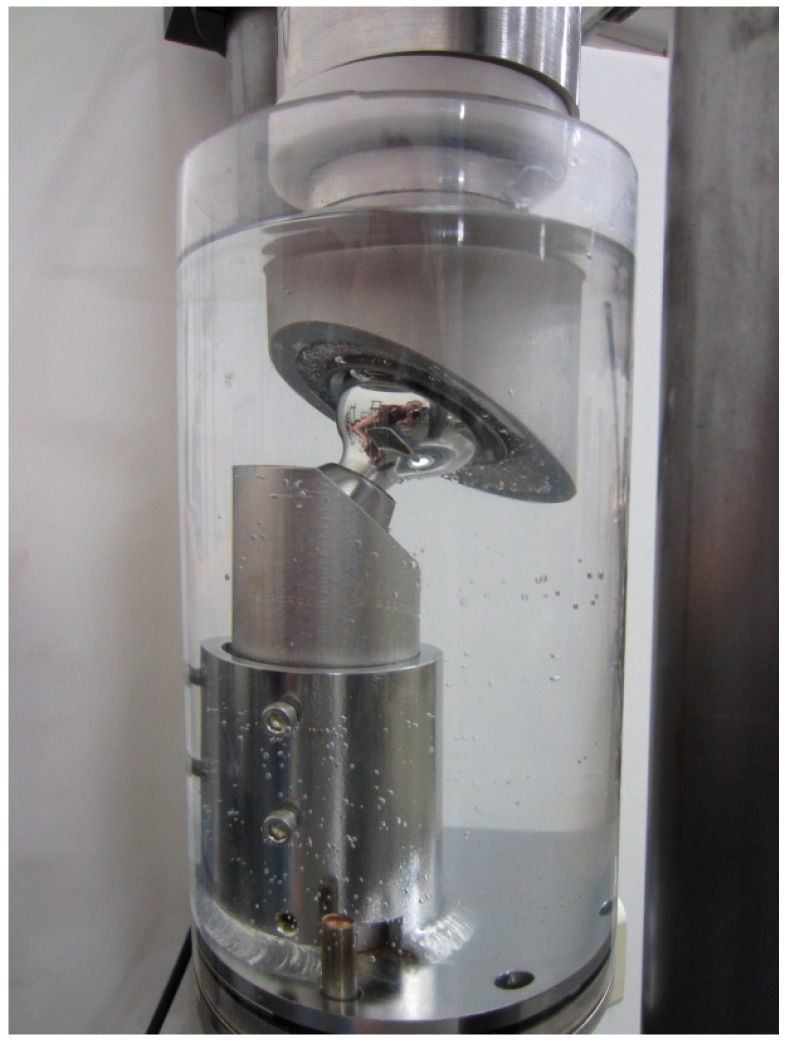
Test setup for corrosion evaluation via ISO 7206-6.

The corrosion potential of the reverse hip system was evaluated using an extended fatigue test. Specifically, the reverse hip system was dynamically tested via ISO 7206-6: 1992. Six hip stem assemblies were oriented at 10° adduction and 9° flexion such that the prescribed load was applied non-parallel to the plane of the neck as designated within the standard. The cementing level was the transection level of the femur as indicated by the superior edge of the porous coating. A custom load application fixture was manufactured to hold the acetabular component at the respective angles. The load applicator was mounted to a self-centering mobile bearing to allow for horizontal travel. The specimen was loaded into the test fixture, and the test chamber was filled with a 0.9% saline solution. The solution temperature was heated to and maintained at 37 °C, as shown in Figure 2. A sinusoidal load profile was applied in load control with a range between 534 and 5340 Newtons (120–1200 lbs. of force) at 10 Hz; the test was terminated at 50 million cycles. 

**Figure 3:**
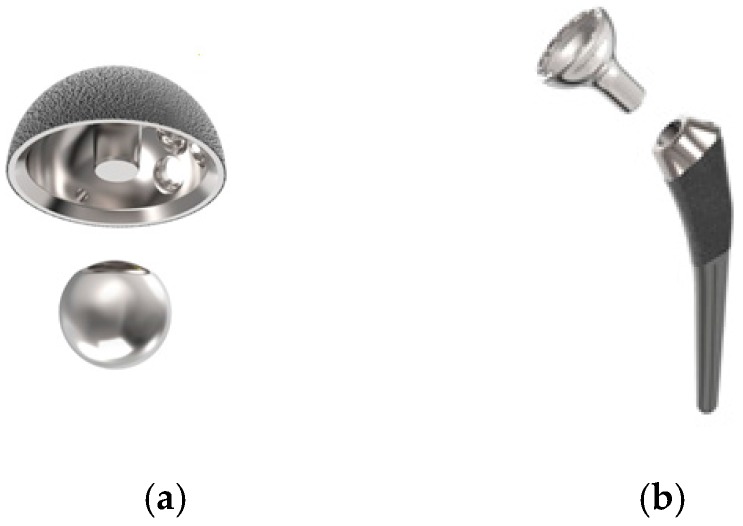
Tapers visually evaluated for corrosion: Acetabular Taper (**a**) and Femoral Taper (**b**).

The stems, femoral cups, acetabular cups, and acetabular balls were inspected after testing for evidence of failure and the tapers were evaluated for evidence of fretting corrosion. Both the acetabular taper between the acetabular cup and acetabular ball and the femoral taper between the femoral stem and the femoral cup, as shown in Figure 3, were evaluated.

Tapers were evaluated with both visual inspection for corrosion and the force needed to remove the ball from the Morse taper after the testing. The visual fretting corrosion was evaluated with the following scale presented at the ASTM Symposium on Metal on Metal Hip Symposium in May 2010:

1. No visual fretting corrosion
2. Less than 31% of engaged taper surface discolored or dull
3. Less than 31% of engaged taper surface discolored or dull or less than 11% of engaged taper surface has black or dull grey debris, pitting or etch marks
4. Greater than 11% but less than 51% of engaged taper surface has black or dull gray debris, pitting or etch marks
5. Greater than 51% of engaged taper surface has black or dull gray debris, pitting or etch

The static disassembly test, as described in ASTM F2009, was performed in displacement control using a Satec testing machine and generating a force versus displacement curve for each sample. 

### 2.3. Polyethylene Wear Characteristics

To determine the wear characteristics of the reverse hip design, the polyethylene liner was articulated against polished CoCrMo balls at varying angles of inclination and anteversion. Inclination was varied from 20° to 70° and anteversion was varied from 0° to 40°. In addition, tests simulating a physiological gait separation of 2 mm were conducted at optimal and malpositioned acetabular cup angles. The testing medium was a 25 + 2% calf serum solution diluted in deionized water. The testing fluid was first filtered through a 2-micron filter and has a protein mass concentration of not less than 17 g/L. To minimize microbial contamination, the fluid test medium was stored frozen until required for testing. An antimicrobial reagent, 20 mM EDTA reagent and 1% penicillin/streptomycin, was added to the serum solution to discourage contaminant growth.

The acetabular component had an articulating surface attached by its normal immediate backing consisting of PMMA and mounted as described in ISO 14242 and the acetabular ball was placed on the device as normal. The femoral cup with the polyethylene liner was mounted to the femoral side of the fixturing via a machined fixture matching the Morse taper characteristics of the femoral stem. The femoral and acetabular components of a test specimen were placed in position in their normal configuration; the test apparatus transmits a specified time-varying force between the components, together with specified relative angular displacements. A control specimen was subjected to the same time-varying force to determine the creep of the test specimen and/or the amount of mass change due to fluid transfer. The test took place in a controlled environment simulating physiological conditions.

Testing was performed on an AMTI 12-station machine which is capable of producing the angular displacements specified ISO 14242-1: 2002. The testing was performed at 1 Hz + 0.1 Hz per the standard recommendations. Each individual specimen was isolated in its own protected environment which prevented third body contamination from the test machine and the atmosphere. The load soak control specimens were subjected to the same uniaxial loading without motion. Specimens were removed from the machine and disassembled at 0.5, 1, 2, 3, 4, and 5 million cycles for gravimetric wear assessment using procedures consistent with ISO 14242-2. 

Additionally, after exposure to 5 million cycles, the system was further tested by adding a 2 mm gate separation for an additional 1 million cycles. The hip simulator vertical loading curve was altered to induce 2 mm of separation between the acetabular ball and the polyethylene insert during the swing phase of the walking cycle and tested under optimal and malpositioned acetabular cup angles for an additional 1 million cycles. The HIT HRS system was compared to both a DePuy and a Zimmer system in a head to head comparison of gravimetric wear debris formation in identical test setups. Additionally, the fluid for the HIT HRS system during the gait separation study was independently analyzed by a third part lab with a priority methodology to determine the chemical composition of the wear debris to evaluate whether metal-on-metal contact was present during the gait separation evaluation. 

## 3. Results

The results of the corrosion analysis and comparative wear debris generation studies are summarized in the following sections.

### 3.1. Corrosion at the Tapers

**Figure 4:**
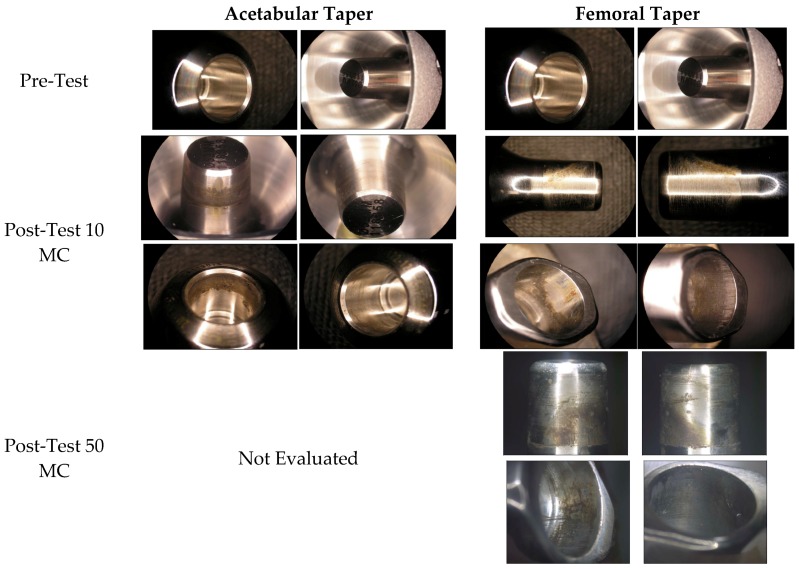
Representative examples of both acetabular and femoral tapers both pre- and post-testing for 10 and 50 million cycles.

The overall surface analysis of the acetabular tapers was performed after exposure to ISO 7206-6 testing in saline with a cyclic loading of 534–5340 Newtons applied at 10 Hz for a total of 50 million cycles. The surface of both the acetabular and femoral tapers were visually evaluated and did not show evidence of surface damage such as gouges, scratches, or structural damage. The grading of the levels of corrosion were scored as a 2 or 3 for the evaluation of the inferior, superior, anterior and posterior faces of all tapers after 10 million cycles indicating discoloration of the taper with minimally pitting or etch marks. Additional evaluation was performed on the femoral taper after a total of 50 million cycles resulting in scores of 2 to 4, indicating between discoloration to some level of pitting and etch marks via the visual analysis scale. Representative examples of the tapers both pre- and post-testing, as shown in Figure 4.

The reverse hip system was additionally evaluated by determining the disassembly forces of the Morse taper connections. The following table summarizes the evaluation of the system post-dynamic testing after 10 million cycles and 50 million cycles, as shown in Table 1.

### 3.2. Polyethylene Wear Characteristics

A comparative analysis was performed between the subject device and two conventional hip arthroplasty systems. For the reverse hip system, the articulating surfaces of the UHMWPE inserts became smooth and burnished during testing. The wear proceeded in a linear manner for all specimens, with similar overall wear rates for all acetabular cup angles. After wear testing to 5 million cycles, the hip simulator vertical loading curve was altered to induce 2 mm of separation between the acetabular ball and the polyethylene insert and tested under optimal and malpositioned acetabular cup angles for an additional 1 million cycles. Head to head wear testing of the reverse system was compared to two commonly known conventional systems, the DePuy Pinnacle and the Zimmer Trilogy. Specifically, the DePuy system consisted of a 28 mm acetabular ball, a 52 mm Pinnacle Duofix HA shell, and a Pinnacle Marathon 28 mm liner. The Zimmer system consisted of 28 mm Versys acetabular ball, a 52 mm continuum shell, and a 28 mm longevity polyethylene liner. The HIT HRS consisted of a 26 mm ball, a 52 mm acetabular cup, and a 26 mm highly crosslinked polyethylene liner. All three systems utilized highly crosslinked polyethylene inserts. The results are summarized in Table 2.

Additionally, a third part laboratory evaluated the testing fluid for the HIT HRS device in order to determine the chemical composition of the wear debris. Using a proprietary evaluation method, it was determined that the particulate composition of the HIT HRS wear debris is polymeric in nature with no detectable metal contamination.

## 4. Discussion

The goal of this study was to evaluate the potential clinical application of a reverse total hip arthroplasty. The reverse hip system evaluated in this study consists of an acetabular component (acetabular cup and articulating ball), a femoral stem, and a polyethylene lined femoral cup. The geometry of the reverse hip prosthesis includes two tapers: the first connecting the acetabular ball and acetabular shell and the second taper connecting the femoral cup and femoral stem. As validated by the testing, the overall surface analysis of the tapers after exposure to ISO 7206-6 testing (534–5340 Newtons, 10 Hz) for a total of both 10 million and 50 million cycles did not show evidence of surface damage such as gouges, scratches, or structural damage. There was no significant difference in either the disassembly force or the level of corrosion on the connecting surfaces when comparing the tapers at either 10 million cycles or 50 million cycles of dynamic fatigue. Since clinical literature has cited that taper disassembly forces of 16,000 Newtons or higher are typically associated with clinically problematic fretting corrosion [6], the levels of disassembly force noted even after 50 million cycles does not raise initial concerns. There was a difference seen between disassembly forces of the acetabular and femoral tapers. Further studies would need to be performed to evaluate the root cause for this difference. 

Additionally, an analysis of the reverse hip system compared with two conventional hip arthroplasty systems was performed. In order to evaluate the reverse hip design for the potential for generation of wear debris, the device was exposed a robust testing regime including 5 million cycles of traditional testing alone with additional testing of 2 mm microseparation on the swing phase of the walking cycle for an additional 1 million cycles. The results of this study show that the wear of the polyethylene liner in the reverse hip system was substantially equivalent to other systems using highly crosslinked polyethylene for the first 5 million cycles. Further challenging of the HIT HRS design with a 2-mm gait separation increased wear debris production as expected but to levels still within acceptable limits. Specifically, the average mass loss over the 1 million cycle gait separation phase of the test was 7.03 mg in total. Assuming the thickness loss is uniform over the entire spherical surface of the polyethylene liner, thickness loss over ten years can be estimated as a polyethylene liner thickness loss of 70 microns. Visual inspection of the liners at various time points showed no evidence of backside wear. Additionally, the edges and rim of the polyethylene liners showed no evidence of edge wear. 

The samples were also evaluated for evidence of subluxation which would manifest in the presence of metal debris. Visual evaluation of both the femoral and acetabular cup showed no signs of contact. The surfaces were free from scratch marks or any other signs of marring of the metal surfaces. 

In summary, the evaluation of the wear generation and subluxation potential of the reverse hip system tested showed the following:

- Wear debris equivalent to other systems using highly crosslinked polyethylene
- Wear debris composition was polymeric only indicating no metal-on-metal contact
- Polyethylene liners showed no signs of back side wear
- Polyethylene liners showed no signs of edge wear

The results demonstrate that the wear properties of the reverse hip system are equivalent to DuPuy’s and Zimmer’s respective conventional systems. Additionally, malposition of the acetabular component did not increase debris generation. Finally, the reverse hip system did not exhibit signs of edge wear even after a total of 6 million cycles of testing with a malpositioned acetabular component and a 2-mm gait separation. The results of this testing provide a firm foundation for further clinical investigation of the reverse total hip arthroplasty.

## 5. Conclusions

We performed extensive biomechanical testing of a novel reverse hip arthroplasty system. The overall surface analysis of the tapers after exposure to ISO 7206-6 testing for a total of both 10 million and 50 million cycles did not show evidence of surface damage such as gouges, scratches, or structural damage. The wear of the polyethylene liner in the reverse hip system was very low even in this challenging test, demonstrating wear properties equivalent to both DuPuy’s and Zimmer’s respective conventional systems. In addition, the reverse hip system did not exhibit signs of edge wear even after a total of 6 million cycles of testing with a malpositioned acetabular component and a 2-mm gait separation. The results of this testing provide a firm foundation for further clinical investigation of the reverse total hip arthroplasty.

## Figures and Tables

**Table 1:** Taper disassembly forces after dynamic fatigue testing where 16,000 N of force or greater is indicative of clinically relevant corrosion.

Condition	Femoral Taper Mean + Standard Deviation (Newtons)	Acetabular Taper Mean + Standard Deviation (Newtons)
10 million cycles dynamic fatigue	12,957 + 1797	3429 + 347
50 million cycles dynamic fatigue	12,506 + 774	4329 + 443

**Table 2:** Wear generation of the reverse hip system versus two conventional systems under normal and gait separation conditions.

Acetabular Cup Placement Inclination/ANTEVERSION	Mean Wear Rate (mg/Million Cycle) No Gait Separation for 5 MC			Mean Wear Rate (mg/Million Cycle) 2 mm Gait Separation for 1 MC		
	HIT HRS	DePuy	Zimmer	HIT HRS	DePuy	Zimmer
45°/0°	1.60 ± 0.45 (*n* = 6)	Not Tested	Not Tested	7.03 ± 0.39 No edge wear (*n* = 3)	Not Tested	Not Tested
20°/0°	1.30 (*n* = 1)	7.09 (*n* = 1)	1.67 (*n* = 1)	6.31 No edge wear (*n* = 1)	14.63 Slight edge wear (*n* = 1)	6.31 Slight edge wear (*n* = 1)
70°/0°	1.71 (*n* = 1)	6.32 (*n* = 1)	1.27 (*n* = 1)	7.08 No edge wear (*n* = 1)	13.16 Slight edge wear (*n* = 1)	6.81 Slight edge wear (*n* = 1)
20°/40°	1.02 (*n* = 2)	Not Tested	Not Tested	5.71 No edge wear (*n* = 1)	Not Tested	Not Tested
70°/40°	1.27 (*n* = 2)	Not Tested	Not Tested	5.76 No edge wear (*n* = 1)	Not Tested	Not Tested
